# A novel brain-enriched E3 ubiquitin ligase RNF182 is up regulated in the brains of Alzheimer's patients and targets ATP6V0C for degradation

**DOI:** 10.1186/1750-1326-3-4

**Published:** 2008-02-25

**Authors:** Qing Yan Liu, Joy X Lei, Marianna Sikorska, Rugao Liu

**Affiliations:** 1 Neurobiology Program, Institute for Biological Sciences, National Research Council of Canada, Ottawa, Ontario, K1A 0R6, Canada; 2 Faculty of Medicine, University of Ottawa, Ottawa, Canada; 3 Department of Anatomy and Cell Biology, University of North Dakota, School of Medicine, 501 N. Columbia Road, Grand Forks, ND 58202, USA

## Abstract

**Background:**

Alterations in multiple cellular pathways contribute to the development of chronic neurodegeneration such as a sporadic Alzheimer's disease (AD). These, in turn, involve changes in gene expression, amongst which are genes regulating protein processing and turnover such as the components of the ubiquitin-proteosome system. Recently, we have identified a cDNA whose expression was altered in AD brains. It contained an open reading frame of 247 amino acids and represented a novel RING finger protein, RNF182. Here we examined its biochemical properties and putative role in brain cells.

**Results:**

RNF182 is a low abundance cytoplasmic protein expressed preferentially in the brain. Its expression was elevated in post-mortem AD brain tissue and the gene could be up regulated *in vitro *in cultured neurons subjected to cell death-inducing injuries. Subsequently, we have established that RNF182 protein possessed an E3 ubiquitin ligase activity and stimulated the E2-dependent polyubiquitination *in vitro*. Yeast two-hybrid screening, overexpression and co-precipitation approaches revealed, both *in vitro *and *in vivo*, an interaction between RNF182 and ATP6V0C, known for its role in the formation of gap junction complexes and neurotransmitter release channels. The data indicated that RNF182 targeted ATP6V0C for degradation by the ubiquitin-proteosome pathway. Overexpression of RNF182 reduced cell viability and it would appear that by itself the gene can disrupt cellular homeostasis.

**Conclusion:**

Taken together, we have identified a novel brain-enriched RING finger E3 ligase, which was up regulated in AD brains and neuronal cells exposed to injurious insults. It interacted with ATP6V0C protein suggesting that it may play a very specific role in controlling the turnover of an essential component of neurotransmitter release machinery.

## Background

Alterations in multiple biological pathways contribute to the development of a sporadic Alzheimer's disease (AD). Amongst these are excessive oxidative stress and insufficient antioxidant defenses, disrupted calcium homeostasis, altered cholesterol synthesis, inappropriate hormonal and growth factor signaling, chronic inflammation, aberrant re-entry of neurons into the cell cycle and, especially, altered protein processing, folding and turnover. The later abnormalities lead to β-amyloid peptide production and senile plagues development, tau hyperphosphorylation and neurofibrillary tangles (NFTs) formation [1,2]. Collectively these changes contribute the loss of synapse, neuronal death and ultimately brain atrophy and dementia characteristic of this disease. However, the scope and complexity of these changes are such that the etiology of sporadic AD still remains elusive.

Recent advances in molecular biology have introduced new, high-throughput tools for the analysis of differential gene expression in complex diseases such as AD. They allow simultaneous overviews of the changes in gene expressions or protein levels for multiple cellular pathways. The most commonly used technology for the assessment of gene expression changes in postmortem brains is the DNA microarray [3-7]. However, this method requires prior knowledge of gene sequences and cannot be applied as a discovery tool for novel transcripts. Furthermore, the expression levels of low abundance genes cannot readily be assessed by DNA microarray hybridization, as reliable results are usually obtained only for genes that are expressed in high or moderate levels. This is a significant limitation as many transcripts expressed preferentially in the brain (e.g., neurotransmitter receptors and their regulatory factors) are present at very low levels [8,9]. Recently, we employed a subtractive hybridization and RNA amplification method to enrich and isolate rare and novel transcripts from AD brains [10]. Using this approach, we have isolated more than 200 genes, which are deferentially expressed, amongst these was a novel brain-enriched sequence that not only was up regulated in AD brains, but also in neuronal cells subjected to injuries.

Here we have described the cloning and characterization of this gene, which encodes a RING finger domain containing protein, resembling an ubiquitin E3 ligase and designated RNF182. We have established that RNF182 can stimulate E2-dependent polyubiquitination *in vitro *and identified an interaction between RNF182 and ATP6V0C. This interaction facilitates the degradation of ATP6V0C via the ubiquitin-proteosome pathway. Overexpression of RNF182 in N2a cells accelerated cell death and it's downregulation reduced cells' response to injurious insults.

## Results

### RNF182 is a novel RING finger-containing transmembrane protein

**Figure 1:**
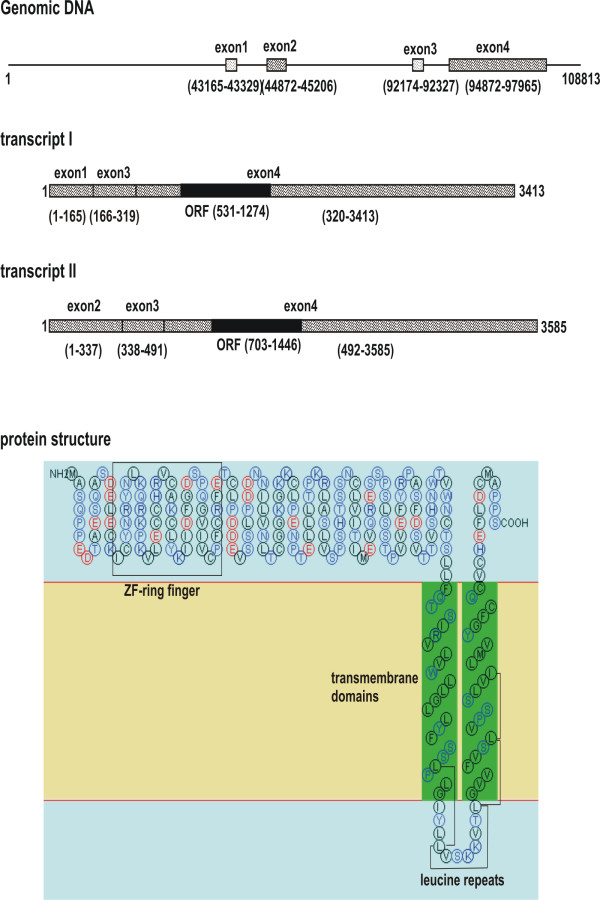
**A schematic representation of the human RNF182 gene, transcript and protein structures**. Solid lines refer to introns or non-transcribed genomic DNA. Hatched bars represent exons. The RNF182 gene (GenBank AL138718) contains four exons. The open reading frame (black bar) and 3' untranslated region of both transcripts are solely encoded by exon 4. Structural motifs of the encoded protein were predicted by ExPASy tools.

We have isolated a 300 bp cDNA fragment, 360nh, by subtractive hybridization using a pooled mRNA population from AD brains as a "tester" and the first strand cDNAs from a pooled age-matched control brains as a "driver" [10]. BLAST searches revealed that this fragment showed a significant sequence identity with human genomic clone RP11-127P7 on chromosome 6 (GenBank AL138718). No matching EST or mRNA for this 360nh sequence was found in GenBank. Genome BLAT analysis indicated that there exists a mRNA sequence (GenBank AK090576) that appeared to be transcribed from the same region of genomic DNA on chromosome 6. This mRNA contains an open reading frame (ORF) encoding a protein with a RING finger domain, dubbed RNF182. Although this mRNA had a 5' end sequence matching the genomic sequence upstream of where 360nh was derived, it did not contain the 360nh sequence. We speculated that the 360nh sequence might be on an alternatively spliced exon of the same gene. We, therefore, used a forward primer on the 5' end of 360nh and a reverse primer within the coding region of RNF182 to amplify any alternative transcript sequence from first strand cDNA synthesized from human brain mRNA. Sequence analysis of the resulting PCR fragment indicated that genomic fragment AL138718, indeed, contained a gene of four exons giving rise to two alternatively spliced transcripts by swapping exons 1 and 2 (Fig. 1). Both transcripts contain the same ORF, thus encoding the same protein of 247 amino acids, with calculated molecular mass of 27.4 kDa. The 360nh sequence was located in exon 2, which constitutes part of the 5' untranslated region of transcript II. Protein sequence comparison revealed that human RNF182 is highly homologous to those of rodents', with 98% and 97% sequence identity to mouse and rat, respectively. The predicted primary structure of this protein contained a typical C3HC4-type RING finger domain between amino acids C20 and C67. There are two putative transmembrane helices located at the C-terminus, spanning amino acids 178 to 200, 212 to 234, respectively. In addition, the primary sequence of RNF182 also suggested four leucine repeats between amino acids 197 and 225. These repeats are within the two transmembrane domains, but do not correspond to a leucine zipper (Fig. 1).

### The expression patterns of RNF182

**Figure 2:**
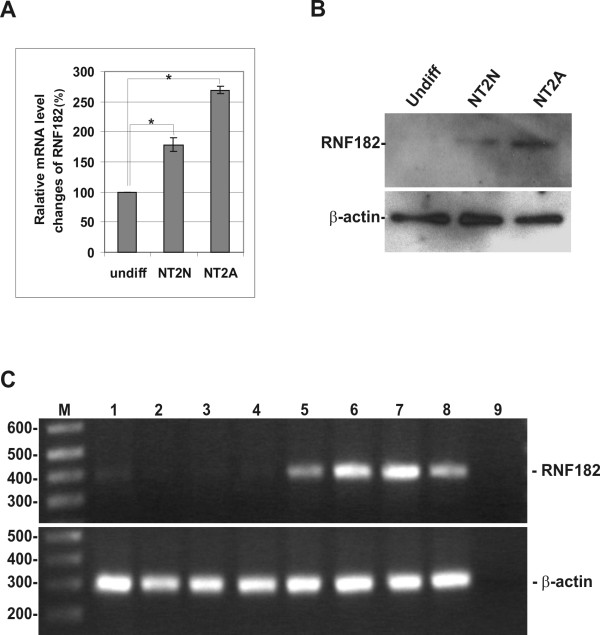
**Expression pattern of RNF182 gene**. **A**. Changes in mRNA levels of RNF182 transcript during RA-induced differentiation of NT2 cells were determined by quantitative RT-PCR. The samples were measured against the cDNA of undifferentiated NT2 cells as a control, set at 100%. Percentage of each sample was calculated by 100x 2^-ΔCt^, where ΔCt is the cycle number difference between test sample and the control sample. undiff – undifferentiated NT2 cells (control), NT2N – NT2 neurons, NT2A – NT2 astrocytes. The experiments were performed in triplicate. Asterisks indicate a significant difference (ρ < 0.05; ANOVA, followed by Bonferronic test). **B**. Changes in RNF182 protein levels were determined by Western blotting with anti-RNF182 antibody using 100 μg/lane of total cellular protein. The Western blotting of β-actin was shown as loading control. **C**. Ethidium bromide stained agarose gel of RT-PCR products amplified from the coding region of RNF182 (top panel) and β-actin (bottom panel) from various mouse tissues: lane M – molecular size marker, lane 1 – kidney, lane 2 – skeletal muscle, lane 3 – liver, lane 4 – heart, lane 5 – cortex, lane 6 – hippocampus, lane 7 – cerebellum, lane 8 – spinal cord, lane 9 – negative PCR control.

RNF182 is a weakly expressed gene, not detectable by Northern blotting. Quantitative RT-PCR analysis indicated that the gene was up regulated during retinoic acid (RA) – induced differentiation of human NT2 cells. The increased level of RNF182 transcripts II was detected in both NT2 neurons and NT2 astrocytes (Fig. 2A). This was further confirmed by Western analysis using anti-RNF182 antibody (Fig. 2B). Next, we analyzed the tissue distribution of RNF182 by semi-quantitative RT-PCR using a primer pair from the coding region of the gene (Fig. 2C). A single band of 395 bp product was detected in the mouse cortex, hippocampus, cerebellum and spinal cord, but not in heart, liver, kidney or skeletal muscle, indicating that RNF182 was a brain-enriched gene.

**Figure 3:**
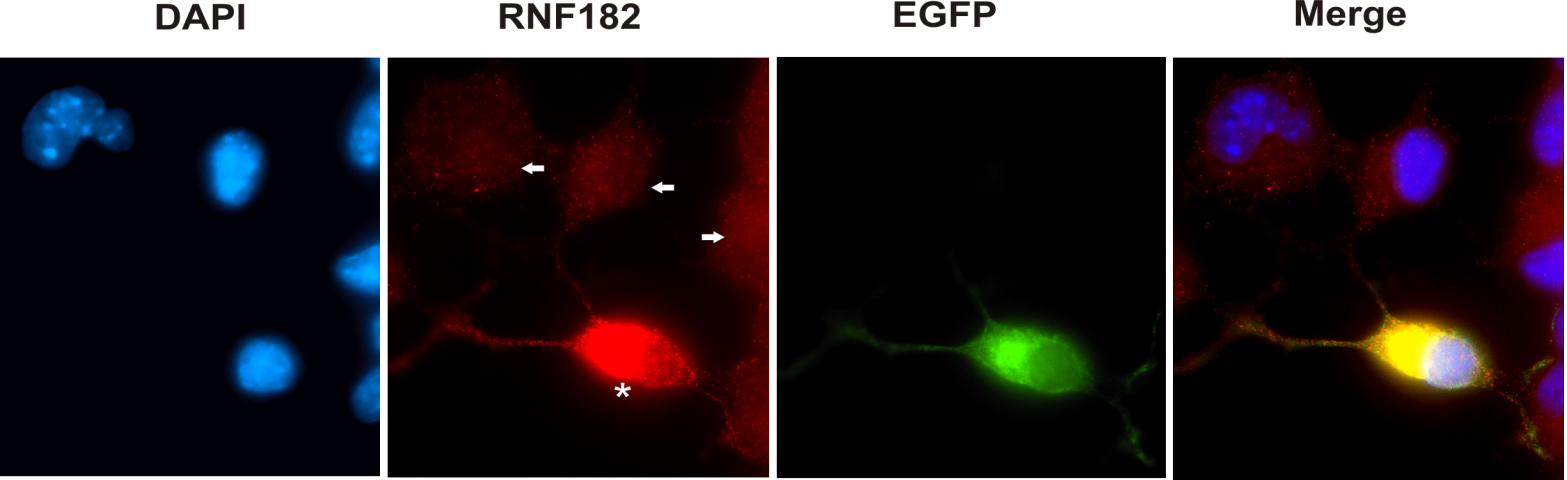
**Subcellular localization of RNF182**. N2a cells were transiently transfected with pEGFP-RNF182 plasmid DNA. Cells were fixed and stained with anti-RNF182 antibody. Cy3-conjugated anti-rabbit IgG was used to detect the specific immunostaining. The nuclei were stained with DAPI and viewed with a Zeiss Axiovert 200 M × 63 fluorescence microscope. Arrows indicate non-transfected cells. Asterisk represents a transfected cell.

To examine the cellular localization of RNF182, we sub-cloned the coding region of RNF182 into the pEGFP-N1 vector where EGFP was fused to the carboxy terminus of RNF182. The plasmid construct was transiently transfected into N2a cells to allow fluorescence detection. The same cell culture was also stained with anti-RNF182 antibody. The non-transfected cell population demonstrated a speckled distribution of endogenous RNF182 protein in the cytoplasm (Fig. 3 panel RNF182, arrows). Fluorescent microscopy of transiently transfected N2a cells revealed a strong signal of the recombinant RNF182 protein throughout the cytoplasm in both differentiated and undifferentiated cells (panel EGFP). No fluorescent signal was observed in the nuclei. Transfected cells showed much stronger staining of RNF182 (Fig. 3 panel RNF182, asterisk), representing a combined signal of both endogenous and exogenous RNF182 protein.

### RNF182 is up regulated in AD brains and in NT2 neurons subjected to injuries

**Figure 4:**
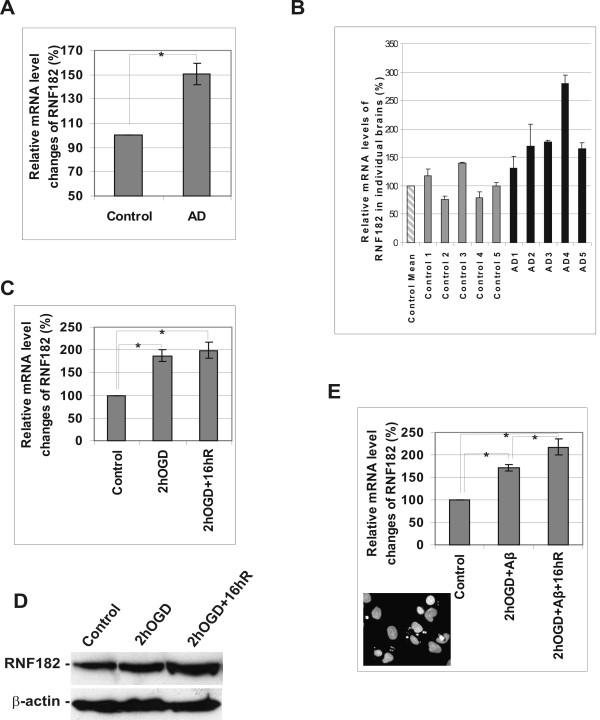
**Upregulation of RNF182 in AD brains and in NT2 neurons treated the cell death-inducing stresses**. **A**. Changes in mRNA levels of RNF182 transcript in control and AD brains were determined by qRT-PCR. The cDNA samples were prepared from pooled mRNA of 4 AD and 5 age-matched control subjects. The value of the control sample was set at 100%. The percentage of the AD sample was calculated by 100x 2^-ΔCt^, where ΔCt is the cycle number difference between the AD sample and the control sample. The experiments were performed in triplicate. Asterisk indicates a significant difference (ρ < 0.005; t-test). **B**. Changes in mRNA levels of RNF182 transcript in individual control and AD brains were determined by qRT-PCR. The cDNA samples were prepared from mRNA of 5 AD and 5 age-matched control subjects. The qRT-PCR results were calculated against the average result (control mean) of the control samples, set at 100%. Percentage of each sample was calculated by 100x 2^-ΔCt^, where ΔCt is the cycle number difference between each sample and the control mean. The experiments were performed in triplicate. **C**. Changes in mRNA levels of RNF182 transcript in NT2 neurons treated with OGD and OGD with 16 h recovery were determined by qRT-PCR. The samples were measured against the cDNA of untreated NT2 neurons as a control, set at 100%. Percentage of each treated sample was calculated by 100x 2^-ΔCt^, where ΔCt is the cycle number difference between treated sample and the control sample. The experiments were performed in triplicate. Asterisks indicate a significant difference (ρ < 0.05; ANOVA, followed by Bonferronic test). **D**. Changes in RNF182 protein levels in NT2 neurons treated with OGD and OGD plus 16 h recovery were determined by Western blotting with anti-RNF182 antibody using 120 μg/lane of total cellular protein. The Western blotting of β-actin was shown as loading control. **E**. Changes in mRNA levels of RNF182 transcript in NT2 neurons treated with OGD plus 20 μM β-amyloid peptide and OGD plus 20 μM β-amyloid peptide with 16 h recovery were determined by qRT-PCR. The samples were measured against the cDNA of untreated NT2 neurons as a control, set at 100%. Percentage of each treated sample was calculated by 100x 2^-ΔCt^, where ΔCt is the cycle number difference between treated sample and the control sample. The experiments were performed in triplicate. Asterisks indicate a significant difference (ρ < 0.05; ANOVA, followed by Bonferronic test). Insert: Nuclear morphology of OGD plus Aβ treated cells was examined under an Olympus B x 50 fluorescence microscope after fixing and staining the cells with DAPI.

Since the RNF182 was found in the subtracted cDNA library containing genes potentially up regulated in AD brains, we re-examined these changes by qRT-PCR analysis (Fig. 4A) of the RNA pools used to construct the original AD and control cDNA libraries [10]. These results were subsequently confirmed using 10 individual brain samples from a tissue bank (Table 1). As shown in Fig. 4B the RNF182 transcript level was consistently higher in AD brain in comparison to the age-matched controls. We also analyzed the RNF182 expression level in post-mitotic NT2 neurons subjected to oxygen and glucose deprivation (OGD), which has been previously reported to trigger neuronal cell death [11]. Here, initially the cells were subjected to 2 h ODG treatment during which 10–15% of cells lost viability, followed by a 16 h recovery period, at the end of which the cell death reached 35–40%. The RNF182 mRNA was significantly up regulated after the OGD treatment (Fig. 4C) and the change in protein level was verified by Western blot analysis (Fig. 4D). These NT2 neurons were insensitive to 20 μM β-amyloid peptide alone, however, when the peptide was added to the culture medium during the OGD and re-oxygenation treatment approximately 55–60% cells died of apoptosis (Fig. 4E, insert). The expression level of RNF182 was doubled after this treatment (Fig. 4E). Taken together, our results indicated that RNF182 was up regulated not only in neuronal cells subjected to the cell death inducing injuries, but also in AD brains where neurodegeneration had become evident.

**Table 1:** Description of brain samples used for qRT-PCR analysis of RNF182

**Patient**	**Sex**	**Age**	**Postmortem**	**Pathology**
Control 1	Male	89	9 hours	Normal
Control 2	Male	64	8 hours	Normal
Control 3	Male	63	8 hours	Normal
Control 4	Male	80	9.5 hours	Normal
Control 5	Male	95	11 hours	Normal
AD 1	Female	84	8 hours	Probable AD, according to CERAD
AD 2	Male	77	6 hours	Senile dementia of AD type
AD 3	Female	81	7.25 hours	Definite AD, possible multi-infarct dementia
AD 4	Male	74	6 hours	Senile changes of AD type
AD 5	Male	N/A	6 hour	Moderate senile changes of AD type, dementia

### Overexpression of RNF182 triggers cell death and its downregulation reduces cell death caused by OGD in N2a cells

**Figure 5:**
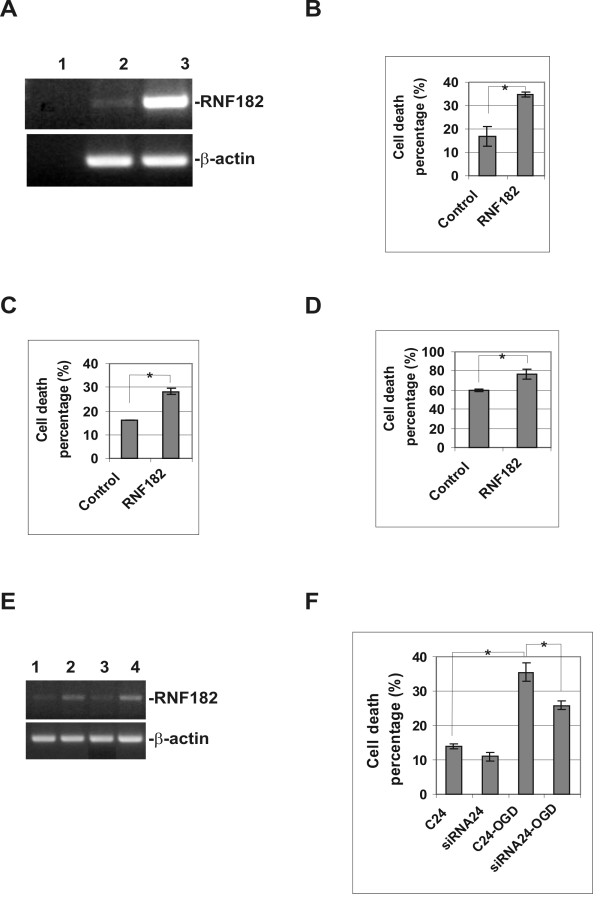
**Cellular levels of RNF182 modulate the rates of cell death**. N2a cells were transiently transfected with RNF182/pcDNA3.1/myc-his plasmid or mouse RNF182 on-target plus smart pool siRNAs. Cells were collected for total RNA extractions 24–48 h after transfections. Trypan Blue exclusion assay was performed 24 h after transfection or 16 h after a 7 h OGD treatment of the siRNA transfected samples. **A**. Over expression of RNF182 mRNA was assessed by RT-PCR. In: lane 1 – negative PCR control, lane 2 – mock transfection, lane 3-transfection with RNF182/pcDNA3.1/myc-his plasmid. **B**. Percentage of cell death before and after transfection. Bars represent the percentage of cell death in the population (mean ± SEM from 3 independent experiments performed in duplicate). Asterisk indicates a significant difference (ρ < 0.005; t-test). **C**. Percentage of cell death before and after transfection with transfection reagent removed 6 h after transfection. Bars represent the percentage of cell death in the population (mean ± SEM from 3 independent experiments performed in duplicate). Asterisk indicates a significant difference (ρ < 0.005; t-test). **D**. Percentage of cell death of the control and 24 h transfected samples subjected to a 7 h OGD treatment. Bars represent the percentage of cell death in the population (mean ± SEM from 3 independent experiments performed in duplicate). Asterisk indicates a significant difference (ρ < 0.005; t-test). **E**. Assessment of the siRNA silencing efficiency. RNA samples were collected 24 and 48 h after transfection with siRNAs. Down regulation of RNF182 mRNA was analyzed by RT-PCR. In: lanes 1 and 3 – 24 and 48 h after transfection with RNF182 siRNAs, lanes 2 and 4 – 24 and 48 h after transfection with non-targeting pool negative control siRNAs, respectively. **F**. Percentage of cell death 24 h after siRNA transfection, with or with out OGD treatment. Bars represent the percentage of cell death in the population (mean ± SEM from 3 independent experiments performed in duplicate). Asterisks indicate a significant difference (ρ < 0.005; t-test). C24 – 24 h after transfection with non-targeting pool negative control siRNAs, siRNA24 – 24 h after transfection with mouse RNF182 on-target plus smart pool siRNAs, c24-OGD – 24 h after transfection with non-targeting pool negative control siRNAs plus OGD treatment, siRNA24-OGD – 24 h after transfection with on-target plus smart pool siRNAs plus OGD treatment.

To better understand the role of RNF182 in neurodegeneration we examined the effects of gene overexpression in N2a cell line. We cloned the coding region of RNF182 into a mammalian expression vector, pEGFP-N1, with a stop codon inserted between the end of the RNF182 and the beginning of EGFP. As a result, a faint green fluorescent signal was observed in the cells transfected with the plasmid construct, but the RNF182 protein was free to perform its routine function without the possible interference of the EGFP. A significant increase in RNF182 mRNA was observed 24 h after transfection (Fig. 5A). The overexpression of RNF182 by itself triggered cell death in N2a cells as compared with mock transfection of empty vector (Fig. 5B). The cell death was not caused by the transfection reagent as removing it did not alter the outcome (Fig. 5C). The subsequent challenge with OGD caused additional loss of cells in both control and the RNF182 transfected cultures (5D). Next, we down regulated the endogenous RNF182 in N2a cells using a mixture of four siRNAs targeting mouse RNF182 gene. The RNF182 transcript was knocked down 24 h after the siRNA transfection (Fig. 5E, lane 1) and still remained low 48 h later (Fig. 5E, lane 3). The transfected cells (after 24 h) were subjected to 7 h OGD treatment and 16 h re-oxygenation in a normal culture chamber and examined for cell viability (Fig. 5F). The results showed that the downregulation of the endogenous RNF182 significantly reduced the percentage of cell death caused by OGD treatment.

### RNF182 exhibits ubiquitin E3 ligase activity

**Figure 6:**
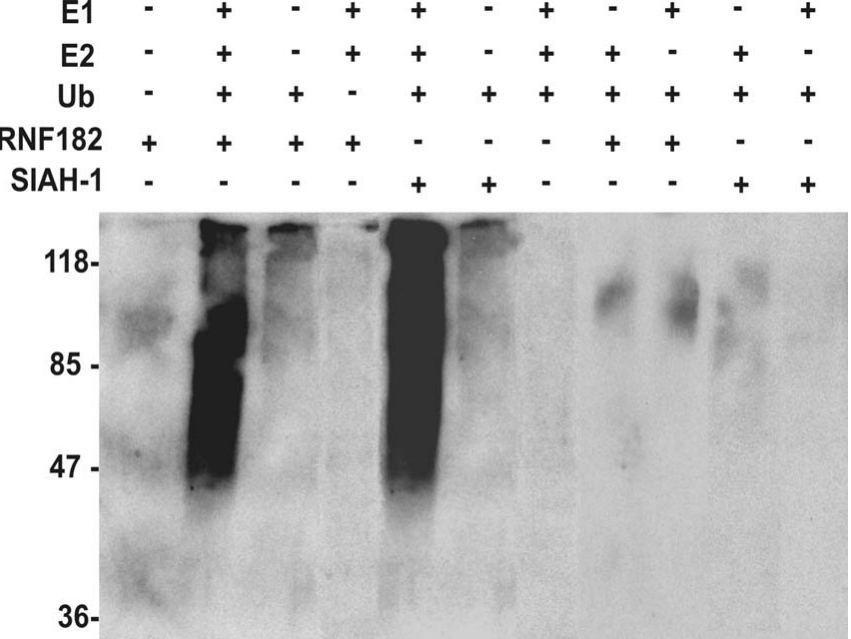
**RNF182 exhibits ubiquitin E3 activity**. His-tagged recombinant RNF182 protein was incubated with or without E1, E2 or ubiquitin, and ubiquitination patterns were detected using an anti-ubiquitin antibody. The GST-SIAH-1 protein was used as a positive control.

Many proteins that contain RING finger domains exhibit ubiquitin E3 ligase activity. Some of these RING finger proteins catalyze substrate-independent, but E2-dependent assembly of multi-ubiquitin chains in reaction mixtures containing ubiquitin, E1, E2 and the RING finger protein itself [12-14]. E3, GST and bacterial proteins from the cell lysate can all serve as potential substrates. With this in mind, we performed an *in vitro *ubiquitination assay to assess whether RNF182 has an E2-dependent E3 ligase activity. An ubiquitination pattern consisting of a high molecular weight smear was obtained from the reactions containing his-tag RNF182 (Fig. 6), whereas none was detected in the absence of RNF182, indicating that RNF182 can function as an E3 ubiquitin ligase. A reaction mixture of E1, E2 and GST-SIAH-1 was used as a positive control. Other controls included RNF182 alone, reactions omitting E3 (RNF182 or GST-SiAH-1), E1, E2 or ubiquitin, all gave negative results. We did not observe apparent auto-ubiquitination of RNF182 by Western blotting of the same blot probed with anti-RNF182 antibody (data not shown), suggesting that the pattern observed in figure 6 was mostly from the ubiquitination of proteins from the bacterial cell lysate. Adding proteins extracted from bacterial clones expressing GST-ATP6V0C or GST alone gave a similar intensity of smears (data not shown). These results confirmed the hypothesis that RNF182 could function as a substrate-independent, E2-dependent E3 ubiquitin ligase.

### The C-terminal domain of RNF182 interacts with ATPV0C

**Figure 7:**
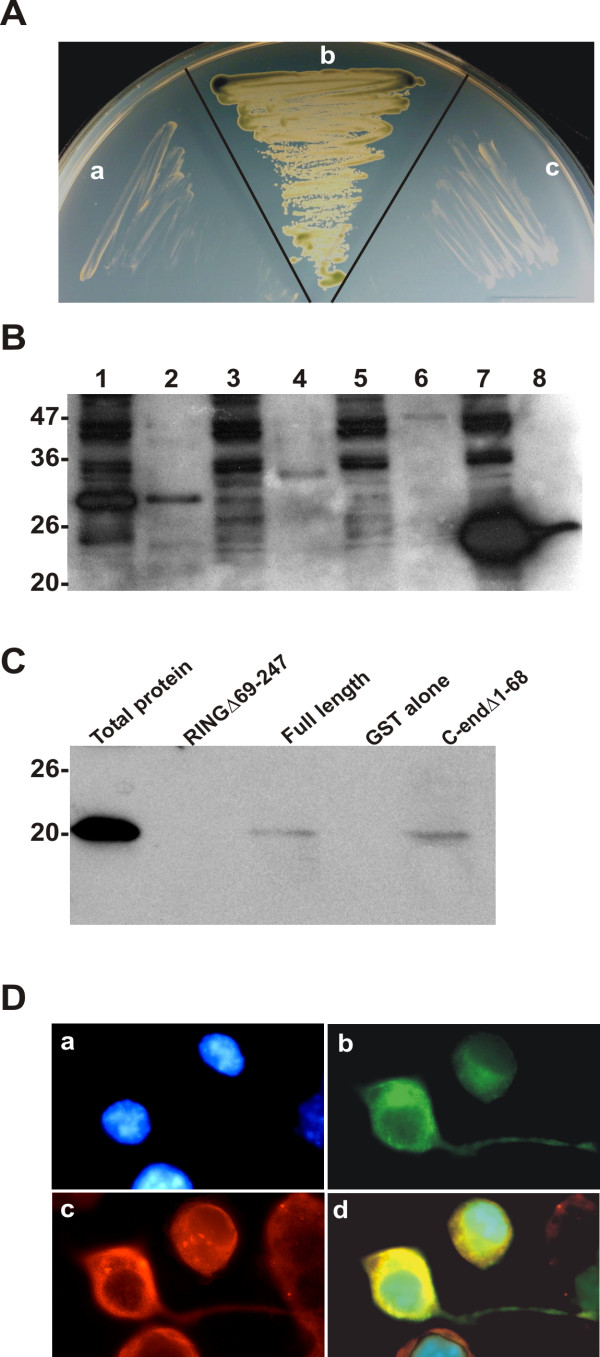
**RNF182 physically interacts with ATP6V0C**. **A**. Interaction between RNF182 and ATP6V0C in yeast two-hybrid system. Empty or RNF182 containing pGBKT7 bait vector and empty or ATP6V0C containing pACT2 library vector were co-transformed into yeast host cells AH109 and plated onto SD/-Trp -Leu -Ade -His +X-α-gal plate. In: a – a negative test of empty bait vector and ATP6V0C; b – a positive test showing the interaction between RNF182 and ATP6V0C; c – a negative test of RNF182 bait plus empty library vector. **B**. Total cellular proteins were extracted from HEK-293 cells co-transfected with flag-tagged ATP6V0C and GST-tagged RNF182 constructs and precipitated with glutathione-sepharose beads as described in the Materials and Methods. The precipitates were separated by 12% SDS-PAGE and transferred onto nitrocellulose membrane. The presence of RNF182 fragments in the complex was revealed by Western blotting with anti-GST antibody. Lanes 1, 3, 5, 7 represent total cellular proteins extracted from cells co-transfected with ATP6V0C and GST-RNF182 RING finger domain, GST-RNF182 C-end domain, GST-RNF182 full length, or GST alone, respectively. Lanes 2, 4, 6, 8 indicate GST fused protein fragments precipitated by glutathione-sepharose beads from cells co-transfected with ATP6V0C and GST-RNF182 RING finger domain, GST-RNF182 C-end domain, GST-RNF182 full length, or GST alone, respectively. **C**. The presence of ATP6V0C in the co-precipitated protein complexes shown in **B **(lanes 2, 4 6, 8) was revealed by Western blotting using anti-flag antibody. In: lane RINGΔ69–247 – GST-RNF182 RING finger domain, lane C-endΔ1–68 – GST-RNF182 C-end domain. **D**. Co-localization of RNF182 and ATP6V0C. N2a cells were co-transfected with flag tagged ATP6V0C and EGFP tagged RNF182 for 24 h. Cells were fixed and stained with anti-flag antibodies. Cy3-conjugated anti-rabbit IgG was used to detect the specific immunostaining. The nuclei were stained with DAPI and viewed with a Zeiss Axiovert 200 M × 40 fluorescence microscope. **a**. DAPI stained nuclei. **b**. EGFP tagged RNF182. **c**. Flag tagged ATP6V0C. **d**. **a**, **b **and **c **overlay.

Since the primary structure of RNF182 contained a RING finger domain and leucine repeats, which often participate in protein-protein interactions, we used yeast two-hybrid screening to identify a potential RNF182 interacting proteins in human brain. Yeast strain AH109 harboring the two-hybrid construct (pGBKT7-RNF182) expressing full length human RNF182 was used to screen a human brain cDNA library. Among 18 clones that displayed Ade/His prototype and β-galactosidase activity, 7 were found to represent a single unique gene encoding human ATPase, H^+ ^transporting, lysosomal 16 kDa, V0 subunit C (ATP6V0C). The interaction between these two proteins was reproducibly reconstructed in the yeast two-hybrid system and it passed all required tests (Fig. 7A). Because the endogenous level of RNF182 was very low, in order to establish the interaction of these two proteins *in vivo*, we fused the RING finger domain, C-terminal domain and full length coding region of RNF182 with GST, which had previously been cloned into the mammalian expression vector pcDNA3.1. The coding region of ATP6V0C was sub-cloned into a pCMV-Tag1 vector carrying a flag tag. To perform the *in vivo *binding assay, HEK293 cells were transiently co-transfected with pCMV-Tag1-ATP6V0C and pcDNA3-GST-RNF182 or deletion constructs. The transfected cells were harvested and the protein extracts were incubated with Glutathione-Sepharose beads. The beads were precipitated by centrifugation, and the samples were boiled and separated by SDS/PAGE. The blot was first probed with anti-GST antibody to ensure that RNF182 protein and the deletion fragments were successfully precipitated by the procedure. We indeed observed fusion proteins of expected sizes after the Western analyses, including GST alone (Fig. 7B). The same protein samples were subsequently probed with anti-flag antibody for ATP6V0C. As shown in Fig. 7C, ATP6V0C bound to full length RNF182 and the C-end domain, but not to the RING finger domain or GST alone. These results confirmed the interaction detected through the yeast two-hybrid assay and also suggested that the RING finger domain did not play a role in this interaction. Next, we examined the sub-cellular localization of RNF182 and ATP6V0C. A EGFP fused RNF182 and flag tagged ATP6V0C were co-transfected into N2a cells. In transfected cells, co-localization of these proteins was detected in a punctuated pattern in the cytoplasmic and perinuclear regions. This is in agreement with the physical interaction detected in the yeast two-hybrid and co-precipitation assays.

### RNF182 facilitates ATP6V0C degradation via the ubiquitin-proteosome pathway

**Figure 8:**
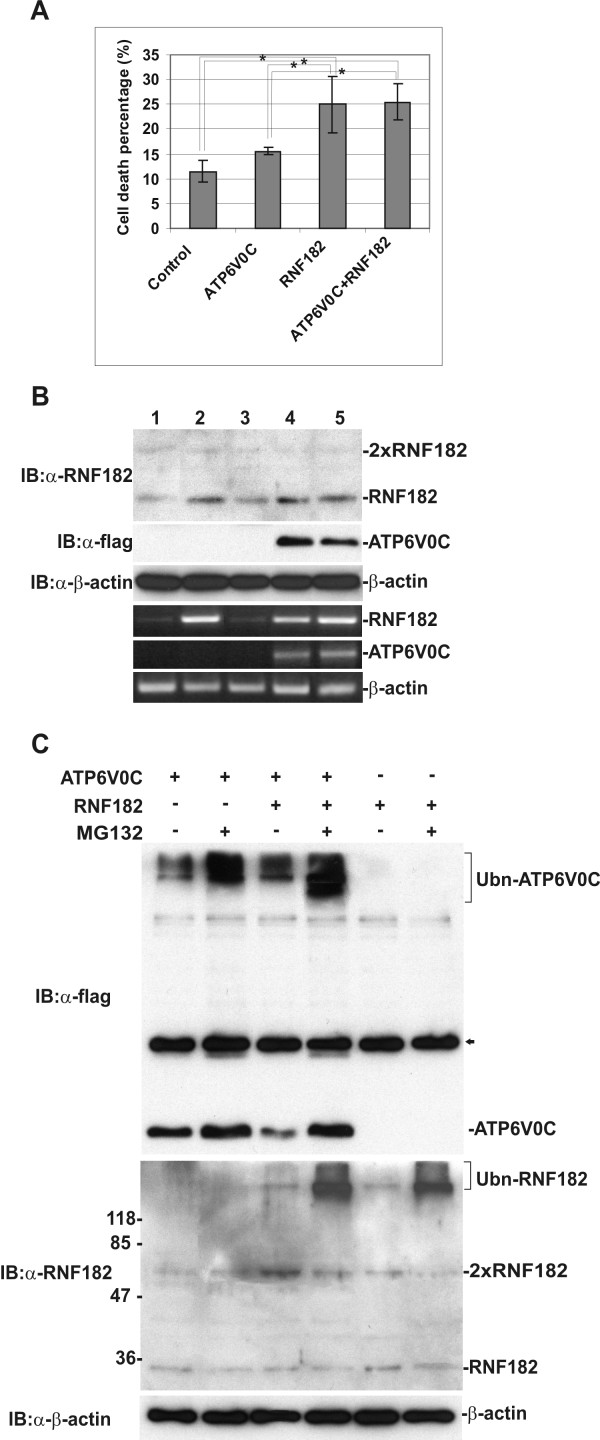
**RNF182 targets ATP6V0C for proteosome degradation**. N2a cells were transiently transfected with empty pEGFP-N1 or pCMV-Tag1 vector alone, pRNF182*EGFP or pCMV-Tag1-ATP6V0C alone, or pRNF182*EGFP and pCMV-Tag1-ATP6V0C simultaneously. Cells were collected for Trypan Blue exclusion assay as well as total RNA and protein extractions 24 h after transfection or treated with 30 μM MG132 for 8 h prior to total RNA and protein extractions. **A**. Over-expression of ATP6V0C in RNF182 transfected cells did not change the percentage of cell death caused by RNF182 over-expression. This figure shows the percentage of cell death before and after transfection. Bars represent the percentage of cell death in the population (mean ± SEM from 3 independent experiments performed in duplicate). Asterisks indicate a significant difference (ρ < 0.05; ANOVA, followed by Bonferronic test). **B**. Western and semi-quantitative PCR analyses of ATP6V0C and RNF182 protein and mRNA levels before and after transfection. IB: indicates primary anti-body used for immmunoblotting. β-actin was used as a loading control for both Western and PCR analyses. In: lane1 – transfection with empty pEGFP-N1 vector, lane 2 – transfection with pRNF182*EGFP, lane 3 – transfection with empty pCMV-Tag1 vector, lane 4 – transfecton with pCMV-Tag1-ATP6V0C, lane 5 – transfection with both pRNF182*EGFP and pCMV-Tag1-ATP6V0C. **C**. Western analysis of changes of ATP6V0C and RNF182 levels before and after transfection followed by MG132 treatment. IB: indicates primary anti-body used for immmunoblotting. Ubn: polyubiquitinated protein. Arrow indicates non-specific bands caused by anti-flag antibody. Western blotting of β-actin was used as a loading control.

To establish the biological significance of the interaction between RNF182 and ATP6V0C, pRNF182*EGFP and pCMV-Tag1-ATP6V0C were co-transfected into N2a cells. Cell death percentage was assessed 24 h after transfection (Fig. 8A). Overexpression of ATP6V0C alone slightly increased cell death rate as compared with mock transfection, but this increase was not statistically significant. Overexpression of RNF182 alone significantly increased the rate of cell death, similar to the results shown in Fig. 5. However, overexpression of both proteins simultaneously in these cells did not hinder nor facilitate the effects of exogenous RNF182 on cell death. These results suggest that it is unlikely that the binding of ATP6V0C to RNF182 inhibits its ability to kill cells, ruling out the possibility that ATP6V0C serves as a RNF182 inhibitor during apoptosis. An alternative explanation would be that the E3 ligase activity of RNF182 facilitates ubiquitination and degradation of ATP6V0C. To test this hypothesis, RNF182 was over expressed with ATP6V0C-flag by transient transfection of N2a cells. Cell lysates were subjected to immunoblotting with anti-RNF182 or anti-flag antibodies. Endogenous RNF182 was often detected as multiple bands, including a major monomeric form with a molecular mass of 27 kDa, and a dimeric form that ran at approximately 54 kDa on SDS-PAGE and occasionally a trimeric form slightly above 80 kDa. The dimeric and trimeric aggregations were more pronounced when total cellular proteins or purified recombinant RNF182 protein had been freeze-dried or been passed through multiple freeze-thaw cycles prior to gel separation. Freshly isolated RNF182 protein appeared mainly as monomers. In cells over expressing RNF182 alone, we detected slightly more RNF182 protein (Fig. 8B, top panel, lane 2); however, this increase was significantly less then that observed at the mRNA level after transfection (Fig. 8B, 4^th ^panel, lane 2). This might be due to the proteosomal degradation of exogenous RNF182 protein since it's overexpression is detrimental to the cells. It is also interesting that in cells over expressing ATP6V0C alone, we observed a dramatic increase of endogenous RNF182 at the transcription level (Fig. 8B, 4^th ^panel, lane 4), which was almost as high as that in the RNF182 transfected cells (Fig. 8B, 4^th ^panel, lanes 2 and 5). This exogenous ATP6V0C-stimulated upregulation of endogenous RNF182 was also reflected at the protein level (compare Fig 8B, top panel, lanes 3 and 4), resulting in a total RNF182 level nearly as high as that of the transfected cells (Fig. 8B, top panel, lanes 2 and 5). These results suggested that there is a positive feedback of ATP6V0C on RNF182 and ATP6V0C may be an ubiquitination substrate of RNF182. This notion was further supported by co-transfection of N2a cells with RNF182 and ATP6V0C, followed by the addition of a potent proteosome inhibitor, MG132, to block the proteosomal degradation of the ubiquitinated proteins. The accumulation of polyubiquitinated ATP6V0C was clearly evident in the inhibitor treated cells as indicated by Western analysis. The steady-state expression of ATP6V0C protein was decreased in the presence of RNF182 as compared with the ATP6V0C level in the absence of RNF182 (Fig. 8C top panel). Furthermore, ATP6V0C expression recovered with the addition of the proteosome inhibitor MG132, despite the overexpression of RNF182, indicating that the RNF182-induced degradation of ATP6V0C was blocked by MG132. These results suggested that RNF182 promoted the degradation of ATP6V0C by the proteosome pathway. In a subsequent experiment, we stripped the above blot and performed another Western analysis with anti-RNF182 antibody. We observed a strong high molecular weight smear of polyubiquitinated RNF182 on the top of the gel in the RNF182 transfected samples treated with MG132, suggesting that a majority of the exogenous RNF182 protein was also degraded through the ubiquitin-proteosome pathway (Fig 8C, middle panel). Therefore, this might offer an explanation as to why we have only detected a slight increase of the RNF182 protein, despite a significant increase of RNF182 mRNA in the transfected cells. MG132 did not cause apparent accumulation of ubiquitinated endogenous RNF182 in the cells transfected with ATP6V0C alone, despite of the fact that ATP6V0C overexpression caused upregulation of endogenous RNF182, suggesting that this physiological increase of RNF182 might be required to degrade unwanted ATP6V0C in the cells.

## Discussion

We have isolated a novel brain-enriched protein, RNF182, which was up regulated in AD brain tissues. Further study of its activities in an NT2 cell model revealed that this gene was barely detectable in undifferentiated NT2 cells, but it clearly expressed in differentiated neurons and astrocytes. Nevertheless, it was still a gene expressed at low abundance as compared with other cellular constituents (such as structural proteins). Consistent with the results obtained from AD brains, treatments of NT2 neurons with OGD or OGD plus β-amyloid peptide caused apparent upregulation of RNF182. Furthermore, overexpression of RNF182 in N2a cells by itself triggered cell death and it's downregulation reduced cell death caused by OGD, suggesting that this gene might have a specific function in brain cells under stress conditions.

One of the structural characteristics of the RNF182 protein is the RING finger domain located at the N-terminus, resembling ubiquitin E3 ligases. Our *in vitro *ubiquitination assay showed that RNF182, indeed, exhibited substrate-independent, E2-dependent ubiquitin ligase activity, which placed this protein in the ubiquitin-proteosome pathway. The most common role of E3 ubiquitin ligase in neurodegenerative diseases is to facilitate the degradation of unwanted, toxic proteins, thus preventing neuronal cell death caused by protein aggregation. For example, synaphilin-1, one of the major components of Lewy Bodies in Parkinson's disease, is a substrate for three RING finger containing E3 ligases [15-17]. Parkin mutations disrupting its E3 activity have been directly linked to autosomal recessive juvenile Parkinsonism [18]. Similarly, a tripartite motif protein, TRIM11, negatively regulates Humanin, a neuroprotective peptide, against AD-related insults, through ubiquitin-mediated protein-degradation pathways [19]. A recent report demonstrates that the upregulation of E3 ligase CHIP (carboxyl terminus of Hsp70-interacting protein) collaborates with Hsp70 to attenuate tau aggregation in AD brains [20]. Quantitative analyses of CHIP in different regions of AD and transgenic mouse brains show that CHIP level is inversely proportional to sarkosyl-insoluble tau accumulation, suggesting that the upregulation of CHIP may protect against the formation of NFTs. Another cytosolic RING finger protein, Dactylidin, is also found up regulated in highly vulnerable regions of AD brains [21]. Although E3 activity of Dactylidin has not yet been demonstrated, these authors speculate that its upregulation in those regions might be related to a putative E3 function. The evidence documented above all seems to indicate a protective function of these proteins due to proteolysis of toxic proteins. However, RNF182 was found to be up regulated during neuronal cell apoptosis and its overexpression alone killed cells, suggesting a role in promoting cell death. This is in agreement with the recent findings that overexpression of a RING finger protein, SIAH-1, triggers apoptotic cell death in various cell types [22]. These authors also find that accumulation of SIAH-1 protein is promoted by its interaction with a scaffold protein POSH upon receiving of apoptotic stimuli. SIAH-1, in turn, activates the JNK pathway, thereby contributing to the death of neurons and other cell types. The E3 ligase activity is essential for SIAH-1-evoked cell death. Based on our RT-PCR and Western blotting results, endogenous RNF182 was low at both mRNA and protein levels and its upregulation during apoptosis was reflected at both the transcription and translation levels. It is not yet clear whether its contribution to cell death is accomplished through collaborations with other proteins. This is still under investigation in our laboratory. Thus far, we found additional brain proteins interacting with RNF182 and their further characterization might shed new light on the biological significance of these interactions.

The interaction of RNF182 with ATP6V0C is intriguing since ATP6V0C is a multi-functional protein that appears to function at the intersection of a number of biological processes. The vacuolar H+ -ATPase (V-ATPase) is a multi-subunit enzyme present in intracellular membrane compartments such as endosomes, lysosomes, clathrin-coated vesicles and the Golgi complex, where it plays a role in their acidification and maintenance of endocytic and exocytic pathways [23]. Its 16 kDa subunit (ATP6V0C) is a membrane spanning protein that folds into four trans-membrane helices and assembles into a hexamer, forming the membrane proton channel of the enzyme [24]. In addition to its role in V-ATPase, ATP6V0C functions independently to form gap junction complexes and neurotransmitter release channels, playing an important role in neurotransmitter release [25,26]. Based on homology comparison with yeast V-ATPase, the 4^th ^trans-membrane domain of ATP6V0C should be located on the exterior of the proton channel where it could easily interact with adjacent protein trans-membrane domains [27]. Scanning the primary structure of RNF182 revealed two typical trans-membrane helices, which could make this E3 ligase unique and placed it in close proximity with ATP6V0C in the membrane, where other E3s might not have easy access. Our results demonstrated that the physical interaction of these two proteins led to the degradation of ATP6V0C through the ubiquitin-proteosome pathway, making the present findings a new illustration of a novel RING finger protein that targets an essential component of neurotransmitter release machinery. This is in agreement with the report of Chin et al. [28], who demonstrate that the RING finger protein, Staring, targets syntaxin 1 for proteosomal degradation, implying an important role of the ubiquitin-proteosome pathway in the degradation of membrane proteins at the nerve terminals.

## Conclusion

We have isolated a novel Ring finger E3 ubiquitin ligase, RNF182, that is up regulated in AD brain and in neuronal cells subjected to cell death-inducing stresses. It's overexpression in N2a cells by itself triggered cell death. It is unlikely that this killing is directly related to its promotion of ATP6V0C degradation, but it might be related to the interactions of NRF182 with other key signaling proteins that are currently investigated in our laboratory. Since ATP6V0C is a key component of gap junctions and neurotransmitter release channels, and RNF182 is up regulated in AD brains, it would be tempting to speculate that RNF182-mediated ATP6V0C degradation contributes to the pathophysiology of this disease. Further study of the molecular mechanism controlling such degradation of synaptic proteins will undoubtedly enhance our understanding of neurodegeneration in AD.

## Methods

### Cell culture and oxygen-glucose deprivation (OGD) treatment

Human embryonal teratocarcinoma Atera2/D1 (NT2) cells (Stratagene, La Jolla, CA), mouse Neuro-2a (N2a) neuroblastoma cells (ATCC CCL-131) and human HEK 293 cells [29] were cultured in Dulbecco's modified Eagle's medium (Invitrogen, Bethesda, MD) supplemented with 10% fetal calf serum (GCS, Wisent, Inc. St. Bruno, PQ), and 40 μg/ml gentamicin sulfate (Sigma Cell Culture, St. Louis, MO). NT2 cells were differentiated into neurons and astrocytes with all trans-retinoic acid (RA, Sigma, Oakville, ON) according to the method of Pleasure and Lee [30] as described previously [31]. N2a cells were differentiated into neurons by replacing culture medium with DMEM containing 0.5% FBS and 20 μM RA for 3 days.

For OGD treatment, NT2 neurons in T75 flasks were washed once with glucose-free DMEM, and incubated in glucose-free DMEM with 10% FBS for 2 h in a Gas Pak 100 chamber (VWR, Montreal, OC, Canada) as described previously [11]. At the end of the OGD treatment, cells were removed from the chamber and returned to the incubator for 16 h. In a parallel experiment, 20 μM β-amyloid peptide (25–35 Aβ amide, Bachem California, Inc) was added to the culture medium during the OGD treatment and re-oxygenation period. The same OGD treatment was performed with N2a cells except the incubation in OGD conditions was 7 h. Cell viability for both cell lines was assessed by the Trypan Blue (Sigma, Oakville, ON) exclusion assay. Labelled cells were counted using a hemocytometer.

### RNA extraction, RT-RCR and real time quantitative RT-PCR (qRT-PCR)

RNA extraction, first strand cDNA synthesis, and qRT-PCR analysis were performed as described previously [32]. RNA pools extracted from frontal cortex of postmortem human brain samples described previously [33] were used for subtractive hybridization and qRT-PCR. Additional brain samples (Table 1) were obtained from the Human Brain and Spinal Fluid Resource Center (VAMC, Los Angeles, CA), which is sponsored by NINDS/NIMN, National Multiple Sclerosis Society, VA Greater Los Angeles Healthcare System, and Veterans Health Services and Research Administration, Department of Veteran Affairs. To detect the expression level of the RNF182 transcript in brain tissue and NT2 neurons, equal amounts of cDNA (2 ng each) were used with the primers: 360nhF 5' TGCCCGTGTGAGCTAGCA 3' and 360nhR 5' AGAACGGAGATATCCATGGTGAA 3' located in exon 2 of the gene. For semi-quantitative RT-PCR, a 395 bp cDNA fragment within the coding region of RNF182, and the entire coding region of ATP6V0C (468 bp) were amplified from first-stranded cDNA using the primers: 395F 5' TTGTGCCAAATGCCTCTACA 3' and 395R 5' ACGTGCAGTTCCACACAGTC 3', vATPcF 5' ATGTCCGAGTCCAAGAGCGGC 3' and vATPcR 5' CTACTTTGTGGAGAGGATGAG 3', respectively. PCR was performed as follows: 1 cycle at 94°C for 5 min, 30 cycles of 94°C for 45 sec, 60°C for 45 sec and 72°C for 45 sec. In the last cycle, the incubation was extended for 5 min at 72°C. The samples were separated on a 1% agarose gel containing 0.5 μg/ml ethidium bromide and photographed.

### Cloning of the FNR182 transcript

A 300 bp cDNA fragment, 360nh, was isolated by subtractive hybridization using the mRNA population from AD brains as a "tester" and the first strand cDNA from control brains as a "driver" [10]. To amplify the cDNA fragment overlapping with both 360nh and the existing mRNA (acc # AK090576), we used a forward RT-PCR primer 5'TGTTGTGGCCCTTAATCTGAGTGCTG 3' and a reverse primer 5' GATGTTGTTGTCATCGGGCAGGCTAC 3'. The PCR conditions were as described above. The resulting PCR product was cloned into pCR-Blunt II-TOPO vector (Invitrogen, Burlington, ON), subsequently analyzed by DNA sequencing and Genbank searches.

### Plasmids and transient transfections

Human cDNA encoding the full length RNF182 protein was cloned into the pBAD/HisA vector (Invitrogen, Burlington, ON) for his-tag RNF182 protein production. To clone the GST-RNF182 or GST-RNF82 RING finger domain, or the GST-RNF182 C terminal domain into a mammalian vector, we first inserted GST sequences into the HindIII/XhoI site of pcDNA3.1 to form a pcDNA-GST tag vector. The entire coding region of RNF182, the N terminal 68 aa containing the RING finger domain and the C terminal 179 aa were then cloned into the EcoRI and XhoI site of the pcDNA3-GST tag vector, in frame with the GST sequence. The coding region of the ATP6V0C cDNA was cloned into the pCMV-Tag1 vector. These plasmids were transfected into HEK 293 cells for the co-precipitation assay.

Human cDNA encoding full length RNF182 protein was cloned in the pEGFP-N1 vector (Clontech, Palo Alto, CA, USA) with or without a stop codon added between the C-terminus of RNF182 and the EGFP sequence and the pcDNA3.1/myc-his vector (Invitrogen, Burlington, ON) to produce pRNF182*EGFP, pRNF182-EGFP and pcDNARNF182-myc-his constructs, respectively. For RNF182-EGFP localization analysis, N2a cells were plated on poly-lysine-coated cover slips in 6-well plates, at a density of 0.5 × 10^6 ^cells/well, 24 h before transfection. Cells were transfected with 5 μg/well of pRNF182-EGFP plasmid DNA and 15 μl LipofectAmine 2000 reagent (Invitrogen, Burlington, ON) according to the manufacturer's instructions. After 24 h, the cells were stained with anti-RNF182 antibody (dilution1:500 v/v) followed by Cy3-conjugated anti-rabbit IgG. The nuclei were counterstained with DAPI in PBS for 5 min and then mounted in Vectashield mounting medium (Vector laboratories, Burlingame CA, USA). The cells were viewed with a Zeiss Axiovert 200 M fluorescence microscope equipped with a Zeiss AxioCam camera (Zeiss, Midland, ON). The images were captured and analyzed using Zeiss Axiovision 3.1 software. For overexpression analysis, N2a cells were plated in 6-well plates at a density of 0.5 × 10^6 ^cells/well, 24 h before transfection. Cells were transfected with 5 μg pcDNARNF182-myc-his plasmid or pRNF182*EGFP plasmid and 15 μl lipofectAmine 2000 reagent, or co-transfected with 2.5 μg each of the pcDNARNF182-myc-his or pRNF182*EGFP and pCMV-Tag1-ATP6V0C plasmids plus 15 μl lipofectAmine 2000 reagent. Cells were collected for Trypan Blue exclusion assay as well as total RNA and protein extraction 24 h after transfection or treated with 30 μM MG132 (Sigma, Oakville, ON) for 8 h prior to total RNA and protein extraction. For siRNA silencing, the on-target plus smart pool siRNAs were purchased from Dharmacon (Dharmacon, Thermo Fisher Scientific, Inc). N2a cells were plated in 12-well plates at a density of 0.25 × 10^6^cells/well, 24 h before transfection. Cell were transfected with 100 μM mouse RNF182 on-target plus smart pool siRNAs using Dharmafect1 transfection reagent according the manufacturer's instructions. Cells were subjected to 7 h OGD treatment 24–48 h after transfection and collected for Trypan Blue exclusion assay 16 h after re-oxygenation. For co-precipitation analysis, HEK 293 cells were plated in 10 cm plates at a density of 2 × 10^6 ^cells/plate, 24 h before transfection. Cells were co-transfected with 7.5 μg of pcDNA3 plasmid DNA harboring a GST-fused full-length, RING finger or C-end RNF182 cDNA fragment and 7.5 μg of pCMV-Tag1-ATP6V0C plasmid DNA mixed with 45 μl LipofectAmine 2000 reagent. Cells were collected for total protein extraction 48 h after transfection.

### Antibody production and purification

Custom polyclonal antibody (GenScprit, Piscataway, NJ) was produced using synthetic peptide N'-ELLLTPKRLASLVSPSH (identical sequence between human and rodent). The immune serum was purified by immunoaffinity purification using recombinant his-tag RNF182 protein. Briefly, purified his-tag RNF182 protein was separated by SDS-PAGE and electro-blotted onto a nitrocellulose membrane. The Ponceau stained membrane portion containing the RNF182 antigen was excised and subjected to a Western blotting procedure using 2 mL original crude serum. The bound antigen-specific antibody was eluted with 0.1 M Glycine-HCl buffer, pH 2.7. The eluted antibody was neutralized by adding 1/10 volume of 1 M Tris, pH 8.5, concentrated using Amicon Ultra-15 Centrifugal Filter Device (Millipore, Fisher Scientific, Ottawa, ON).

### Protein extraction, Western blotting and co-precipitation

Recombinant His-tag RNF182 protein was purified from Top10 cells using a HiTrap nickel column (Pharmacia Biotech, Baie d'Urfe', QC). The recombinant GST fusion SIAH-1 protein was purified from Rosetta cells harboring a pGEX4T-1/SIAH plasmid (a kind gift of Dr. M. Weissman, NIH, USA) [14]. For total protein extraction from cultured cells, cells were trypsinized and collected by centrifugation. They were washed twice with PBS and lysed with RIPA buffer containing 1X protease Inhibitor cocktail (Roche Diagnostics, Indianapolis, IN). The lysate was vortexed and incubated on ice for 15 min, followed by sonication for 30 sec. In some cases the total cellular protein was freeze-dried and reconstituted in PBS in order to achieve a higher concentration. Western blotting analyses were performed as previously described [34]. The blots were probed with the following primary antibodies: Rabbit polyclonal, affinity-purified anti-RNF182 (1:1000), rabbit polyclonal anti-flag (1:1000, Rockland, Gilbertsville, PA), goat polyclonal anti-GST (1:1000, Amersham Phamacia Biotech, Baie d'Urfe, QC), mouse monoclonal anti-ubiquitin (1:1000), and mouse monoclonal anti-β-actin (1:5000 v/v, both Sigma, Oakville, ON). The antigens were detected using horseradish peroxidase-conjugated secondary antibodies: anti-mouse IgG (1:5000 v/v), anti-rabbit IgG (1:5000 v/v, both from Jackson ImmunoResearch Laboratories, Inc., West Grove, PA) or anti-goat IgG (1:5000, Sigma, Oakville, ON). The antigen-antibody complexes were visualized by enhanced chemiluminescence using an ECL Plus detection kit (Amersham Phamacia Biotech, Baie d'Urfe, QC).

For the co-precipitation assay, flag-tagged ATP6V0C and GST-tagged RNF182 constructs were transiently co-transfected into HEK-293 cells and total cellular proteins were extracted as described above. The extracts were incubated with 200 μl of glutathione-sepharose beads for overnight at 4°C. Beads were precipitated by centrifugation at 10,000 g for 1 min and washed four times with PBST (1% Triton ×-100 in PBS), and samples were boiled in protein loading buffer and separated by 12% SDS-PAGE. The presence of RNF182 fragments and ATP6V0C in the complex was revealed by Western blotting as described above.

### Yeast two-hybrid screening

Human cDNA encoding the full length RNF182 protein was cloned into the pGBKT7 vector (Clontch, Palo Alto, CA, USA) to generate a chimaeric open reading frame encoding the *Gal*4 DNA binding domain and RNF182 protein. This construct was introduced into *Saccharomyces cerevisiae *strain AH109. A single colony containing cells harboring the pGBKT7-RNF182 plasmid was then used to provide host cells for screening a human brain cDNA expression library constructed using the pACT2 vector (Clontech, Palo Alto, CA, USA). The protein-protein interaction was first screened by plating the transformants onto SD/-Trp-Leu-His-Ade selection plates. Positive clones were then re-screened for the presence of β-galactosidase activity to eliminate false interactions. Library plasmids harboring RNF182 interacting proteins were rescued and re-introduced into the RNF182/pGBKT7-containing host cells to further eliminate false interactions. The identity of the cDNA encoding RNF182-interacting protein was revealed by DNA sequencing and database searches.

### *In vitro *ubiquitination assay

Ubiquitination experiments were carried out according to a previously published report [14] with modifications. Thirty microliter, *in vitro *reactions were performed in ubiquitination buffer (50 mM Tris-HCl, pH 7.4, 2.5 mM MgCl_2_, 0.5 mM DTT, 2 mM ATP, 1 mM creatine phosphate) containing 0.5 units of creatine phosphokinase, 750 ng his-tag RNF182 or 1.3 μg GST-SIAH-1 (in the case of positive control reactions), 55 ng E1 (Boston Biochem, Cambridge, MA), 85 ng E2/Ubc5a (Boston Biochem, Cambridge, MA), 10 μg ubiquitin (Sigma-Aldrich, Oakville, ON), and 2 μL of bacterial lysate from Rosetta cells transformed with pGEX-3X. The mixture was incubated at 30°C for 90 min and the reaction was stopped by adding 5X SDS-PAGE loading buffer. The reaction mixture was resolved on 8% SDS-PAGE gel and analyzed by Western blotting using mouse anti-ubiquitin monoclonal antibody (Sigma-Aldrich, Oakville, ON).

## Competing interests

The author(s) declare that they have no competing interests.

## Authors' contributions

QYL participated in the design of the study, analysis and interpretation of the data and preparation of the manuscript. JXL performed most experiments and helped to analyze the data. MS dissected postmortem human control and AD brain tissues and participated in writing of the manuscript. RL helped to analyze the data and contributed to writing of the manuscript. All authors read and approved the final manuscript.
